# Front-End Development for Radar Applications: A Focus on 24 GHz Transmitter Design

**DOI:** 10.3390/s23249704

**Published:** 2023-12-08

**Authors:** Tahesin Samira Delwar, Unal Aras, Abrar Siddique, Yangwon Lee, Jee-Youl Ryu

**Affiliations:** 1 Department of Smart Robot Convergence and Application Engineering, Pukyong National University, Busan 48513, Republic of Korea; 2 Department of Global IT Engineering, Kyungsung University, Busan 48434, Republic of Korea; 3 Department of Spatial Information Engineering, Pukyong National University, Busan 48513, Republic of Korea

**Keywords:** 24 GHz, power amplifier, radar, transmitter front-end, up-conversion mixer

## Abstract

The proliferation of radar technology has given rise to a growing demand for advanced, high-performance transmitter front-ends operating in the 24 GHz frequency band. This paper presents a design analysis of a radio frequency (RF) transmitter (TX) front-end operated at a 24 GHz frequency and designed using 65 nm complementary metal-oxide-semiconductor (CMOS) technology for radar applications. The proposed TX front-end design includes the integration of an up-conversion mixer and power amplifier (PA). The up-conversion mixer is a Gilbert cell-based design that translates the 2.4 GHz intermediate frequency (IF) signal and 21.6 GHz local oscillator (LO) signal to the 24 GHz RF output signal. The mixer is designed with a novel technique that includes a duplex transconductance path (D_TP_) for enhancing the mixer’s linearity. The D_TP_ of the mixer includes a primary transconductance path (P_TP_) and a secondary transconductance path (S_TP_). The P_TP_ incorporates a common source (CS) amplifier, while the S_TP_ incorporates an improved cross-quad transconductor (I_CQT_). The integrated PA in the TX front-end is a class AB tunable two-stage PA that can be tuned with the help of varactors as a synchronous mode to increase the PA bandwidth or stagger mode to obtain a high gain. The PA is tuned to 24 GHz as a synchronous mode PA for the TX front-end operation. The proposed TX front-end showed an excellent output power of 11.7 dBm and dissipated 7.5 mW from a 1.2 V supply. In addition, the TX front-end achieved a power-added efficiency (PAE) of 47% and 1 dB compression point (OP_1_dB) of 10.5 dBm. In this case, the output power is 10.5 dBm higher than the linear portion of the response. The methodologies presented herein have the potential to advance the state of the art in 24 GHz radar technology, fostering innovations in fields such as autonomous vehicles, industrial automation, and remote sensing.

## 1. Introduction

In the fast-paced realm of contemporary technology, where the boundaries of wireless communication and radar systems continue to expand, the role of the transmitter (TX) front-end stands as a linchpin in the quest for performance optimization, efficiency enhancement, and the pursuit of greater reliability [1]. The front-end of a radio frequency (RF) TX is responsible for generating and transmitting the radar signal [2]. Ensuring the integrity of this signal is essential for accurate target detection and measurement. Any distortions or noise in the signal can lead to erroneous radar measurements [3]. Also, radar systems often operate within specific frequency bands. The front-end design must ensure that the TX operates within regulatory constraints and does not interfere with other RF systems or receive unwanted interference. The front-end design of an RF TX in radar applications is of critical importance as it plays a fundamental role in the overall performance and functionality of the radar system.

**Figure 1:**
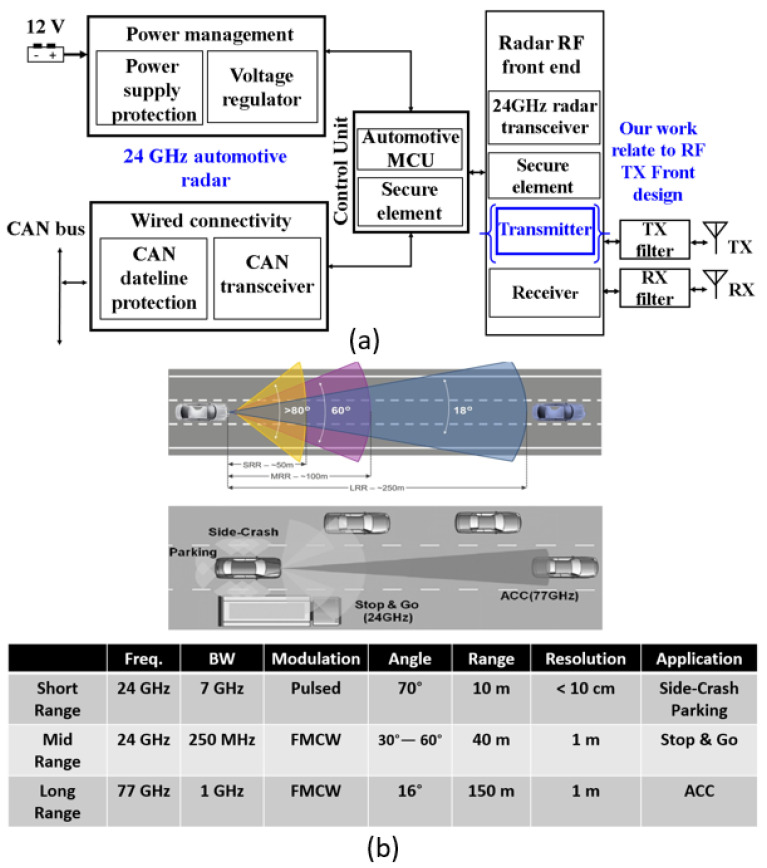
(**a**) A 24 GHz radar block diagram; (**b**) radar application.

The 24 GHz frequency band has emerged as a beacon of innovation in radar technology. Its unique attributes, characterized by a delicate balance between signal propagation and absorption, have rendered it an optimal choice for a multitude of radar applications. This frequency range is a critical domain for applications such as vehicular radar, industrial sensing, and vital safety systems, where precision in target detection and a robust performance in challenging environments are paramount. A 24 GHz TX front-end is a crucial component in automotive radar systems, playing a pivotal role in generating and delivering the radar signal for applications such as adaptive cruise control and collision avoidance. A 24 GHz TX front-end typically consists of several functional blocks that together enable the generation and transmission of a radio signal at 24 GHz. A 24 GHz TX front-end comprises essential components, such as a stable VCO, power-efficient PA, pulse modulator, compact directional antenna, and optional beamforming elements, collectively designed to generate and deliver precise radar signals in automotive applications. Figure 1 shows a block diagram of a typical automotive radar and its application, respectively.

In principle, the 24 GHz complementary metal-oxide-semiconductor (CMOS) TX front-end is based on a TX configuration consisting of an up-converter mixer and a PA block. In practice, the development of a 24 GHz building block of CMOS TX front-end design is quite challenging [4]. In theory, the disadvantage of the CMOS has lowered the power consumption [5]. Due to high fabrication temperatures and the quality of the silicon substrate, manufacturing devices on a flexible substrate is not feasible. The use of a silicon substrate restricts the number of dielectrics that may be utilized, and employing high-dielectric-constant gate insulators is difficult. Also, the most obvious inherent disadvantage of CMOS technology is its low breakdown voltage. Because PAs and T/R switches are subjected to a high-power signal at the end of the transmitter, a low breakdown voltage has a direct impact on their performance and dependability.

The CMOS RF front-end block, in particular the design of the PA, is difficult in radar applications [6,7,8]. The most common causes of distortion and power consumption at the RF front-end are PAs. PAs are frequently used in linear classes to reduce linearity deterioration. However, PAs result in a poor average power efficiency, which leads to concerns about the transceiver battery life. A CMOS up-conversion mixer design is also crucial to drive an outer PA by adjusting the 50 ohm load. The mixer causes a low output impedance, which tends to obtain a low CG, conversion loss, and high power consumption. Thus, an effective research effort is needed to explore the novel design of the PA and mixer [9,10,11,12,13,14].

### 1.1. Related Literature

In prior research, it has been demonstrated that the CMOS is a potential medium for manufacturing RF circuit blocks in the low-gigahertz spectrum. A high-performance CMOS front-end for applications exceeding 20 GHz, however, has yet to be described. The previous research shows [15] a design of an RF TX front-end explanation with a minimal voltage and power for 2.4 GHz ZigBee applications. In [16], the author designs a CMOS TX front-end with adaptive TX equalization in a 0.13 μm CMOS process at 3.5 GHz. The author, Pieter [17], demonstrates a TX front-end based on digital polar using 65 nm technology. The measurement result shows EVM value percentages of 1.90% for 5 MHz and 6.08% for 946 MHz and 20 MHz bandwidth signals at 2.4 GHz. The work [18] shows an integrated RF TX front-end that addresses the demand for wideband antenna adjustment from 1.5 to 5 GHz. The article [19] presents a simple TX front-end architecture to achieve a highly efficient PAE. The manufactured output has a high PAE of 67.5% and a compact and integrated RF-front structure with a size reduction of 43%. Author Dan [20] describes a W-band TX front-end with an output power of 4 dBm at 77 GHz and OP_1_dB of 2.2 dBm at 85 GHz in a 65 nm CMOS.

The work presented in [21] demonstrates a TX front-end to solve the problem of VCO pulling and crosstalk. The front-end achieves an average output power of 5.3 + 4.8 dBm. However, the TX front-end does not address the issue of the high power consumption and low efficiency of the circuit blocks, which can impact the overall performance of the system. Also, the impact of the nonlinearity of the TX and phase noise of the LO on the EVM is not thoroughly analyzed in the paper. Another author, Shin [22] shows in a paper a front-end that consists of an I/Q up-conversion mixer, a two-stage PA, and an I/Q LO generator circuit. The methods described in the paper focus on the design and implementation of the TX front-end but do not provide details on specific measurement techniques or experimental procedures. The work [23] deals with a 24–28 GHz four-element phased-array transceiver (TRX) front-end (FE) for 5G communications. The proposed TRX FE can be effectively deployed in both base stations and user equipment for 5G communications. However, the paper does not discuss comparisons with other existing TRX FE designs and the potential trade-offs or compromises made in terms of power consumption, area efficiency, or other performance metrics in the design of the TRX FE.

From the other prior research, a high-efficiency TX front-end circuit for 24 GHz FMCW radar applications, incorporating a PA stage and a voltage-controlled oscillator (VCO), is presented in [24]. The paper does not provide a detailed analysis of the performance degradation or reliability concerns that may arise due to the dissipation of energy as heat in the amplifier efficiency. The paper does not address the potential variations in the oscillation frequency and performance degradation of the overall front-end due to the variation in the PA input capacitance. The work in [25] presents a circularly polarized balanced radar front-end topology with a TX leakage canceller, implemented using a printed circuit board and InGaP/GaAs heterojunction bipolar transistor technologies. The proposed circularly polarized balanced radar front-end topology has losses of 6 dB in the transmitting path and 6 dB in the receiving path. The difference between the simulated and measured results for the additional suppression of Tx leakage is 6 dB, which is attributed to process errors. In [26], a high-efficiency 27–30 GHz 0.13 μm BiCMOS TX front-end for SATCOM phased arrays was designed. It includes a system-level analysis for determining key parameters, fully characterized building blocks, and measurement results for large-signal performance. The work does not provide information on the potential drawbacks or trade-offs associated with the measured transducer gain, power consumption, and power-added efficiency of the chip. A 26 GHz TX front-end using a double quadrature architecture, which eliminates the need for an image rejection filter in the mm wave frequency band is shown in the study [27]. The TX achieves a high conversion gain, low distortion, and low power consumption, making it suitable for applications in 5G communications. The study does not mention any comparative analysis or performance evaluation of the proposed TX front-end with existing or alternative solutions. Saito N et al. present a fully integrated transceiver chipset based on the WiGig/IEEE 802.11ad standard for mobile usage, targeting a reduced power consumption [28]. However, it does not provide information about the performance of the transceiver chipset in non-line-of-sight (NLOS) scenarios or in environments with high interference.

Furthermore, in [29], a 64-QAM 60 GHz CMOS transceiver that can transmit 10.56 Gb/s in all four channels defined in IEEE802.11ad/WiGig, achieving a TX-to-RX EVM of −26.3 dB, is shown. It does not discuss any potential drawbacks or trade-offs associated with achieving the high data rate of 28.16 Gb/s in 16 QAM using a four-bonded channel. In the work [30], the author presents the design of a 60 GHz out-phasing TX in a 40 nm bulk CMOS, optimized for a high output power and peak PAE while maintaining linearity. The chip achieves a 500 Mb/s 16 QAM modulation with a 12.5 dBm average output power and 1% average efficiency (PA) at an EVM of 22 dB, with further improvements in average output power and efficiency through mismatch compensation and phase correction. The use of compensation reactances or capacitor banks to improve back-off efficiency and facilitate tunability and on-chip integration is not considered in the work. The impact of mismatch compensation (MC) on the modulated signal measurement results is considered to be very low, but a detailed analysis or quantification of this impact is not provided.

However, as the application horizon broadens, so does the complexity of the challenges. Designing a TX front-end at 24 GHz for radar applications demands meticulous attention to detail and a profound comprehension of the intricate trade-offs that must be negotiated. Among the challenges are signal attenuation in various environmental conditions, interference with other radio systems, and the necessity of real-time and high-precision target detection [18,19,20,21,22]. Tackling these intricacies requires innovative design strategies, advanced signal-processing techniques, and sophisticated analysis tools.

Throughout this study, we focus on a comprehensive analysis of the 24 GHz spectrum for radar applications. Futhermore, we describe the 24 GHz TX front-end circuit. The 24 GHz TX front-end design includes a D_TP_-based up-conversion mixer structure and a PA for the 24 GHz automotive radar in detail.

### 1.2. Main Contributions

The following list summarizes and discusses the main contributions of this paper:

1. A 24 GHz TX front-end circuit is proposed in this work.

2. The proposed circuit includes the integration of a $D_{TP}$-based up-conversion mixer block and a PA.

3. The novelty of the 24 GHz TX front-end design is that the proposed mixer employs a novel $D_{TP}$ technique to increase the linearity. In addition, we present the design of a synchronous mode PA for class AB. PA implementation helps to increase the PA gain and efficiency.

4. The main significance of designing an RF TX front-end is to ensure improvements in power efficiency and linearity for an automotive radar system.

This paper is described in four sections. Section 2 describes the $D_{TP}$-based up-conversion mixer and a PA. Section 2.3 describes the proposed RF TX front-end design. And Section 3 and Section 4 refer to the result analysis and conclusion of this paper.

## 2. TX Front-End Integrated Circuit

### 2.1. CMOS Up-Conversion Mixer for TX

**Figure 2:**
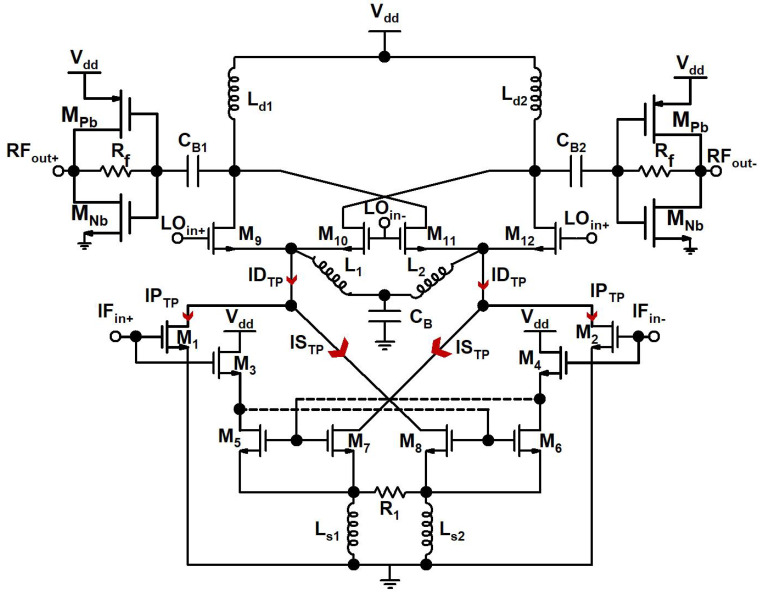
A 24 GHz D_TP_-based up-conversion mixer.

A 24 GHz D_TP_-based up-conversion mixer schematic designed for a TX front-end is shown in Figure 2. Table 1 represents the mixer component values.

The IF input signal is fed and amplified by the I_CQT_ and differential CS amplifier in the D_TP_ stage, which is designed to enhance the linearity and transconductance of the mixer. Transistors M_1_ and M_2_ formed a differential CS amplifier that is a P_TP_ in the D_TP_ stage of the mixer, and the 2.4 GHz IF signal is coupled with the gate node of M_1_ and M_2_. Transistors M_3_–M_8_ and resistor R_1_, which act as feedback resistors and are coupled between source nodes of M_7_ and M_8_, together formed an I_CQT_ that is an S_TP_ in the D_TP_ stage of the mixer, and the IF is also fed at the gate terminals of the M_3_ and M_4_ transistors of I_CQT_. M_3_ and M_4_ transistors are also cross-connected with M_5_–M_7_ and M_6_–M_8_, which are current mirror transistors. In the I_CQT_, the L_s1_ and L_s2_ are source degenerated inductors. The C_1_ bypass capacitor and L_1_ and, L_2_ inductors are connected to the common node of the D_TP_ stage and LO switching stage for better inter-stage matching and to increase the mixer gain. The LO switching stage is designed with M_9_–M_12_ transistors and a 21.6 GHz LO differential input signal is applied at the gate nodes of these transistors. L_d1_ and L_d2_ inductors serve as the load for the mixer’s RF output stage, and the n/pMOS complementary transistors M_Nb_ and M_Pb_ with the R_f_ resistor formed an output buffer for the RF output signal.

**Figure 3:**
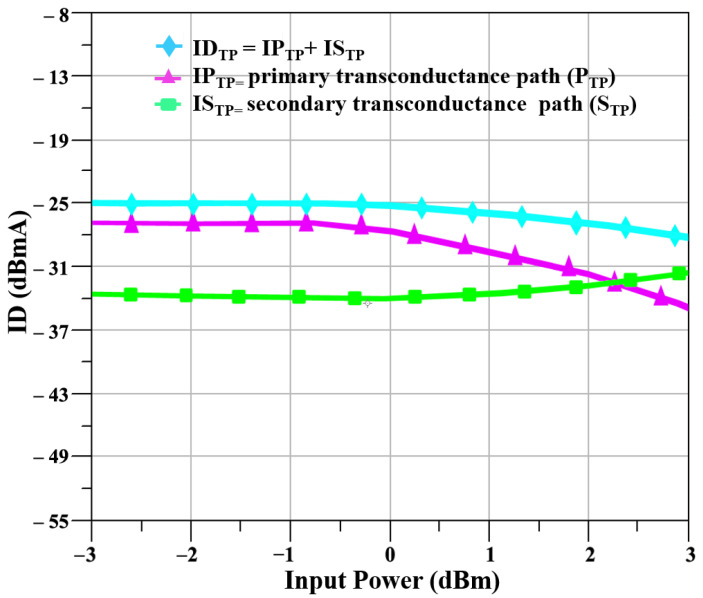
Output currents of D_TP_ stage.

**Figure 4:**
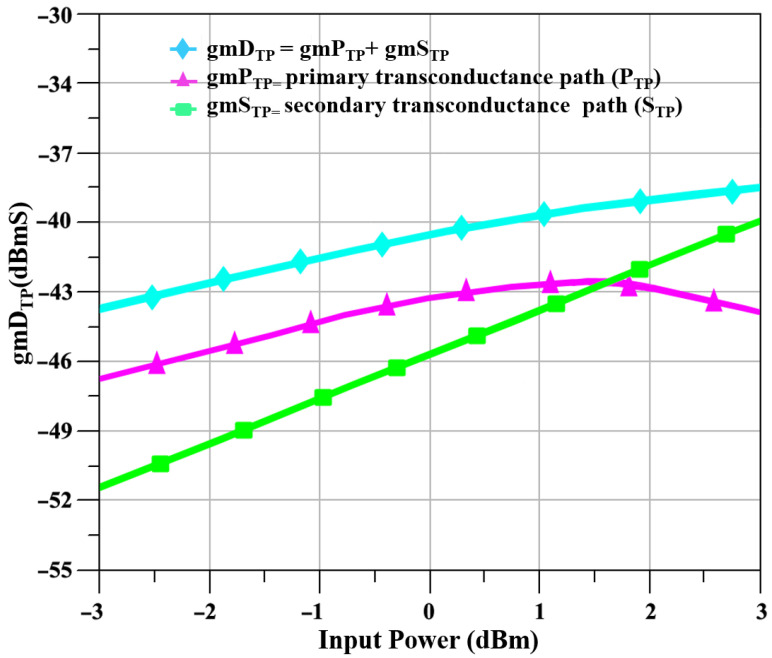
The transconductances of gmP_TP_ and gmS_TP_ along with the total transconductances gmD_TP_ versus input power.

The S_TP_ that is the I_CQT_ is included in the mixer design to enhance the linearity and to increase the transconductance, as at a 24 GHz frequency linearity is the main concern of CMOS technologies, and this linearity issue cannot be solved with only the M_TP_ that is the CS amplifier because of its own limitations. The currents of the IP_TP_ and IS_TP_ of the P_TP_ and S_TP_, respectively, and the total current of the ID_TP_ of the D_TP_ are shown in Figure 3, and it is evident from the figure that the current that the IS_TP_ has a linear relationship with the input power, as the input power is increased to 4 dBm, while the current of the IP_TP_ starts to depict nonlinear behavior when the input power is 1.5 dBm. Similarly, the transconductances for the gmP_TP_ and gmS_TP_ of the P_TP_ and S_TP_, respectively, and the total transconductance of the gmD_TP_ of the D_TP_ are shown in Figure 4. The transconductance depicts a compressive behavior for the P_TP_ and rising behavior for the S_TP_ as the input signal power increases.

In the traditional cross-quad transconductance, the source node of M_5_ and M_6_ shows a similar voltage level and presents a virtual short connection that weighs down the linearity from the transistors to R_1_, the feedback resistor, and the transconductance values show a dependency on the tail current source, which is equal to 1/R_1_. The traditional cross-quad circuit depicts two limitations. The first one is that it creates positive feedback if the load is attached to the drain nodes of the M_3_ and M_4_ transistor through gate drain (C_gd_) and parasitic capacitance, and the second one is at mm wave frequencies, where the parasitic inductances in the traditional cross-quad circuit depict a negative resistance in between source nodes of M_5_ and M_6_. Both these limitations may create instability in traditional cross-quad circuits. In the I_CQT_, these limitations are rectified as M_5_, M_7_, M_6_, and M_8_ current mirror transistors are used, and positive feedback is averted, while the linear output signal is acquired from the drain nodes of the M_7_ and M_8_ transistor.

If the IF voltage signal is considered to be V_if+_ = Acosω_if_+__t, the output currents of the D_TP_ and I_1_ for the P_TP_ from the drain terminal M_1_ transistor and I_8_ for the S_TP_ from the drain terminal M_8_ transistor are shown in Equations (1) and (2), respectively [14].

(1) $$i_{1}=\frac{g_{m1}2A^{2}}{2}+\left(g_{m1}1A+\frac{3g_{m1}3A^{3}}{4}\right)cos\omega_{if+}t+\frac{g_{m1}2A^{2}}{2}cos2\omega_{if+}t+\frac{g_{m1}3A^{3}}{4}cos3\omega_{if+}t+\ldots$$

(2) $$\begin{array}{cc} i_{8}=\frac{M_{7,8}}{\left(M_{5,6}+M_{7,8}\right)}\frac{g_{m8}2A^{2}}{2}+\frac{M_{7,8}}{\left(M_{5,6}+M_{7,8}\right)}\left(g_{m8}1A+\frac{3g_{m1}3A^{3}}{4}\right)cos\omega_{if+}t & \\ +\frac{M_{7,8}}{\left(M_{5,6}+M_{7,8}\right)}\frac{g_{m8}2A^{2}}{2}cos2\omega_{if+}t+\frac{M_{7,8}}{\left(M_{5,6}+M_{7,8}\right)}\frac{g_{m8}3A^{3}}{4}cos3\omega_{if+}t+\ldots & \end{array}$$

where g_m1_ and g_m8_ are the transconductances of the M_1_ and M_8_ transistors, respectively. The total transconductance g_m8_ of the D_TP_ is the summation of the transconductances of the P_TP_ that is g_m_P_TP_ and of the STP that is g_m_S_TP_. The total transconductance g_mt_ and IP_1dB_ is the 1 dB compression point of the mixer and is given in Equations (4) and (5).

When the transconductances of the P_TP_ and S_TP_ add together the total transconductance g_mt_ and 1 dB compression point IP_1dB_ of the designed mixer, they are equal to [14]

(3) $$g_{mt}=g_{m\left(P_{TP}\right)}+g_{m\left(S_{TP}\right)}$$

(4) $$g_{mt}=\left(g_{m1}1+\frac{M_{7,8}}{\left(M_{5,6}+M_{7,8}\right)}g_{m8}1\right)+\frac{3A^{2}}{4}\left(g_{m1}3+\frac{M_{7,8}}{\left(M_{5,6}+M_{7,8}\right)}g_{m8}3\right)$$

(5) $$IP_{1dB}=\sqrt{0.145\left|\begin{array}{cc} \; & \frac{g_{m1}1+\frac{M_{7,8}}{\left(M_{5,6+}M_{7,8}\right)}g_{m8}1}{g_{m1}3+\frac{M_{7,8}}{\left(M_{5,6+}M_{7,8}\right)}g_{m8}3}\\ \; & \end{array}\right|}$$

The R_1_ resistor in the I_CQT_ and the ratio of the current mirror transistor are set to obtain a high linear transconductance, so the I_CQT_ increases the linearity of the mixer. Yet the D_TP_ circuit dissipates more power than the traditional mixer, due to the increment to the second current path, but it also attains a high linearity compared to the traditional mixer.

**Figure 5:**
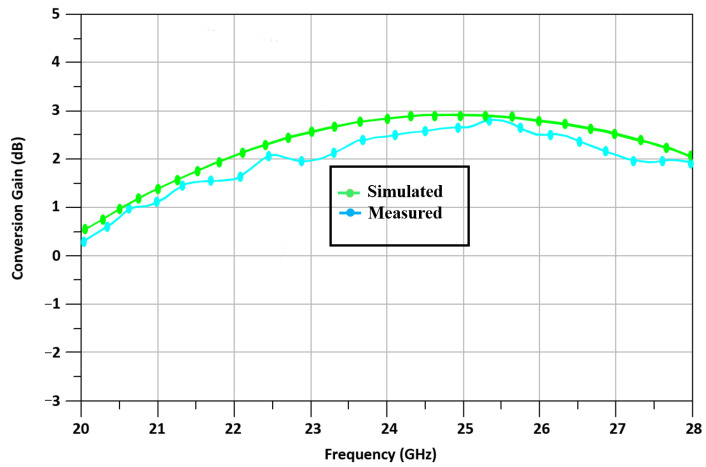
Conversion gain vs. frequency.

Figure 5 shows the up-converted mixer’s CG at 24 GHz. The D_TP_-based up-conversion mixer accomplishes a measured CG of 2.49 dB.

**Figure 6:**
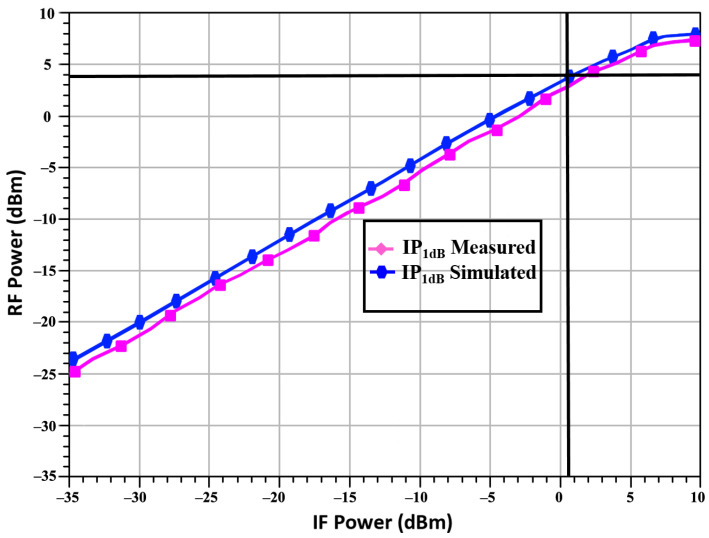
Power of RF output vs. IF input.

Figure 6 shows the linearity of the RF o/p power against the measured IF i/p power. The designed D_TP_-based up-conversion mixer achieved an IP_1_dB equal to 0.9 dBm at 24 GHz. For an easy understanding of the nonlinear characteristics, the IP_1_dB of the simulated result was depicted as 0.9 dBm, and the OP_1_dB was 3.9 dBm. In terms of linearity, the mixer performed well at 24 GHz, within a reasonable frequency range.

### 2.2. CMOS PA for TX

**Figure 7:**
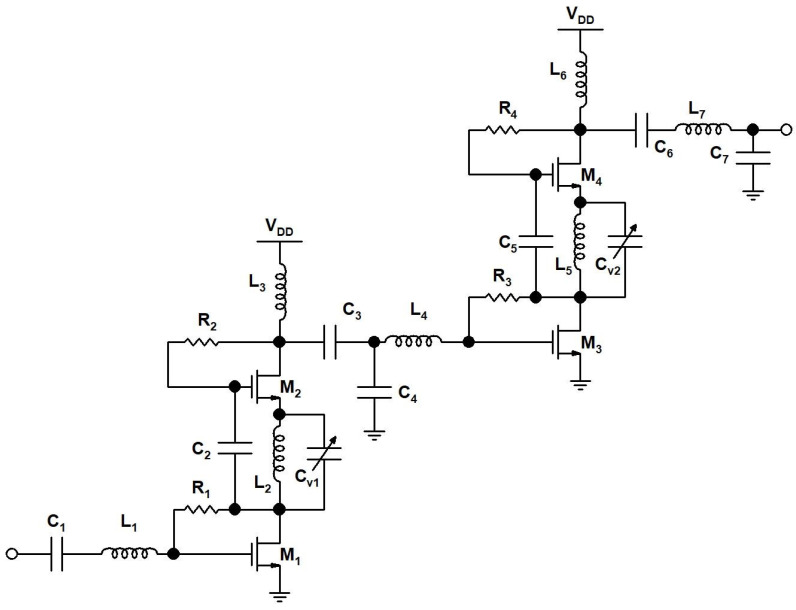
A 24 GHz CMOS PA schematic.

The CMOS PA schematic designed for the TX front-end is shown in Figure 7. The PA in the TX front-end is a class AB tunable two-stage PA that can be tuned with the help of varactors as a synchronous mode to increase the PA bandwidth or stagger mode to obtain a high gain. The PA is tuned to 24 GHz as a synchronous mode PA for the TX front-end operation. The PA’s first stage includes transistors M_1_–M_2_ connected as a cascode structure, C_2_ and C_v1_ capacitors, an L_2_ inductor, and R_1_ and R_2_ resistors, while the second stage includes transistors M_3_–M_4_ connected as a cascode structure, C_5_ and C_v2_ capacitors, an L_5_ inductor, and R_3_ and R_4_ resistors. The M_1_–M_2_ and M_3_-M_4_ transistors’ size widths are 106 μm and 204 μm, respectively. The L_2_ inductor is connected in between the M_1_ transistor drain terminal and M_2_ transistor source terminal in the first stage of the PA, and the L_5_ inductor is connected in between the M_3_ transistor drain terminal and M_4_ transistor source terminal in the second stage of the PA. L_2_ and L_5_ inductors offer a high impedance that helps in the amplification of the RF signal, while L_3_ and L_6_ inductors connected at the M_2_–M_4_ transistors’ drain terminal help in resonating out the drain parasitic capacitors. As a result, the transistor pairs M_1_ and M_3_ and M_2_ and M_4_ both accomplished high gains. The R_1_, R_2_, R_3_, and R_4_ resistors are feedback resistors connected with M_1_, M_2_, M_3_, and M_4_ transistors, respectively, which offer self-biasing and also minimalize the nonlinearity of the transistors and improve the linearity of the PA. The input impedance matching network is implemented with a C_1_ capacitor, L_1_ inductor, R_1_ resistor, and parasitics of M_1_. The inter-stage impedance matching between the first and second stage is implemented using C_3_–C_4_ capacitors, L_3_–L_4_ inductors, and R_3_ resistors; all these components are tuned for maximum RF signal transfer from the first to the second stage of the PA. The PA output impedance is matched to 50 Ohm using matching L_6_ and L_7_ inductors and C_6_-C_7_ capacitors. C_v1_ and C_v2_ are the varactors connected in parallel to the L_2_ and L_5_ inductors, respectively. C_v1_ and C_v2_ are tuned to the same capacitance value to have a synchronous operation of the PA in the TX front-end. Table 2 presents the PA component values.

**Figure 8:**
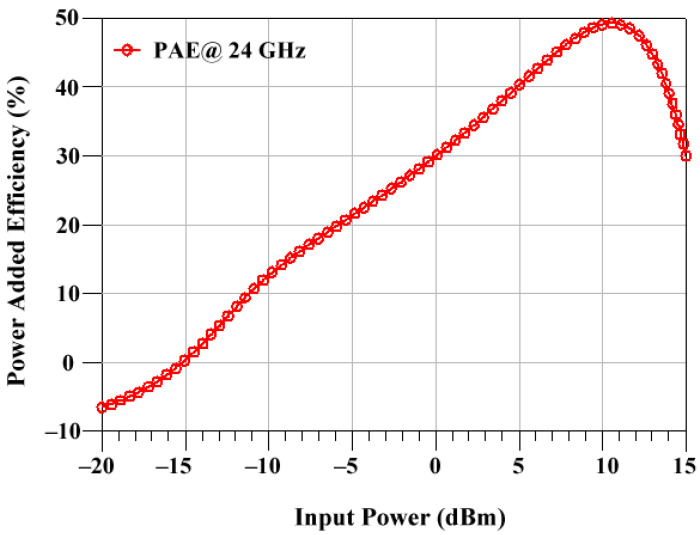
Power-added efficiency of PA.

From Figure 8, we can see the PAE of 47.5 % is achieved at 24 GHz. The radar applications benefit greatly from the PA’s efficiency.

**Figure 9:**
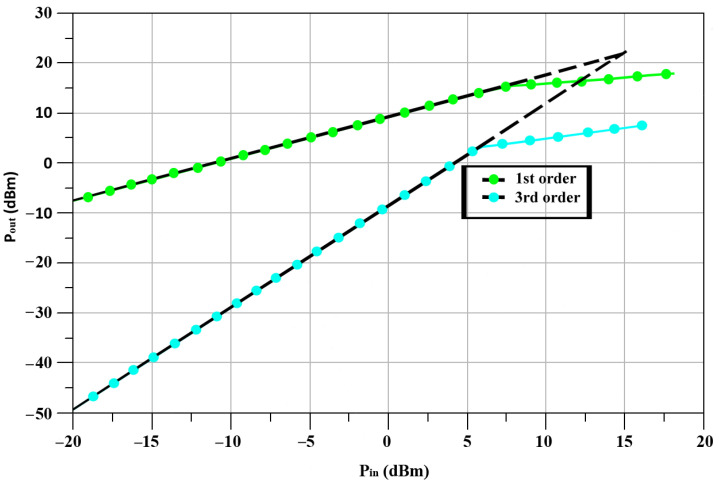
IIP3 and OIP3.

According to Figure 9, the PA obtains a good IIP3 of 14.5 dBm. To obtain such high linearity, self-biased resistive feedback is utilized.

### 2.3. Proposed TX Front-End: Building Blocks and Integration

**Figure 10:**
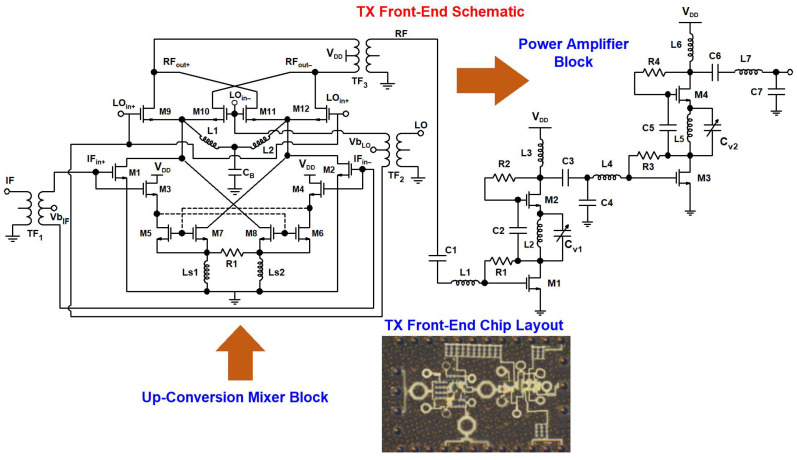
Schematic of a proposed 24 GHz TX front-end and chip layout.

The TX front-end IC fabricated using 65 nm CMOS technology integrates a novel up-converter mixer and novel PA design. In Figure 10, a schematic of a proposed 24 GHz TX front-end and chip layout is shown. The 2.4 GHz IF signal and 21.6 GHz LO signal are translated to a 24 GHz RF signal by the up-converter mixer. The IF and LO are generated off-chip and connected to the TX IC pad. The 2.4 GHz IF signal source is fed to the IF port of the mixer through the transformer TF_1_, which converts the single-ended IF signal to the differential signal and provides a 50 ohm input match. The input IF signal is amplified in the D_TP_ stage. The IF signal is connected to both the P_TP_ and S_TP_ of the transconductance phase. The LO signal source with a 21.6 GHz frequency is coupled with the LO port of the designed up-conversion mixer through the transformer TF_2_, which also provides the 50 ohm matching between the input LO signal source and the LO port of the mixer of the TX. The RF port of the mixer is connected with the final circuit block of the TX front-end, which is a PA, through the transformer TF_3_, which along with the C_1_ capacitor, L_1_ inductor, R_1_ resistor, and parasitics of the M_1_ transistor, acts as a 50 ohm match between the mixer RF port and the input port of the on-chip integrated novel PA. All the transformers are laid out using top metal m9 and m8 metal layers of 65 nm CMOS technology. The IC area is 3.2 mm^2^, including all DC and RF input and output pads.

## 3. Results and Discussion

**Figure 11:**
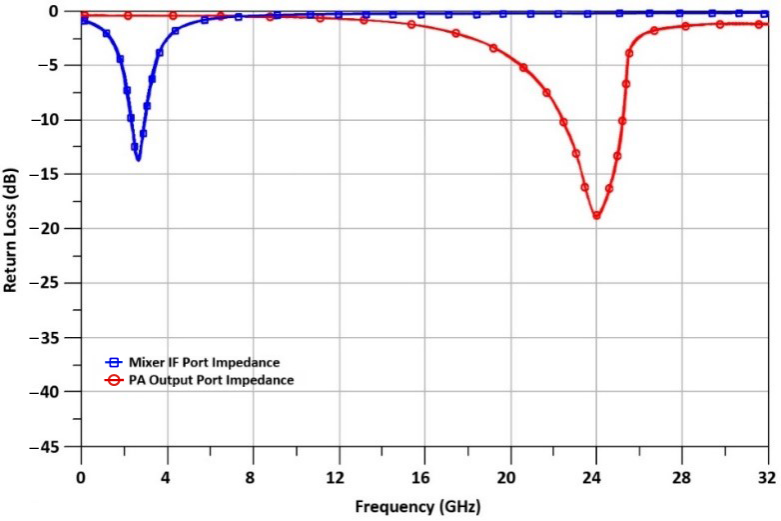
Input IF port and output return loss of the TX front-end.

A 65 nm CMOS process was used to implement the 24 GHz TX front-end. The small signal results of the designed TX front-end are shown in Figure 11. In the following figure, the input return loss (S_11_) of the IF port is shown along with the output return loss (S_22_) of the TX front-end. At 2.4 GHz and 24 GHz, the measured input and output return losses are each −13.2 dB, lower than −10 dB, and well within 50 ohm.

**Figure 12:**
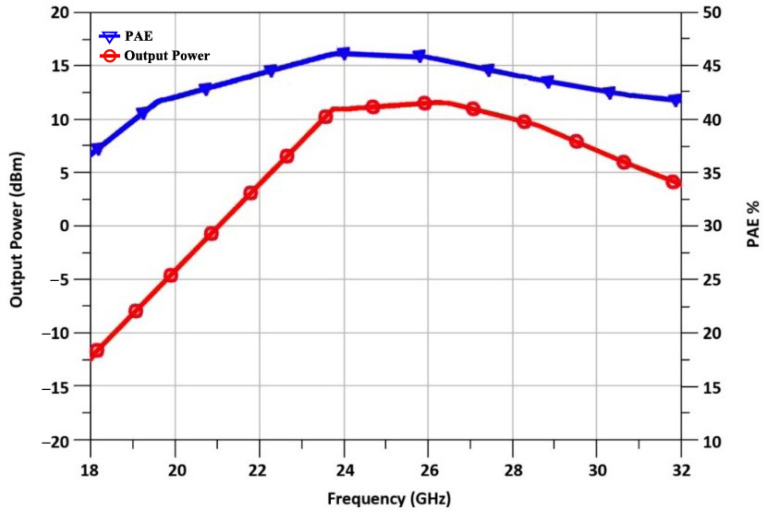
PAE and output power of the designed TX front-end.

Figure 12 depicts the result of the PAE and output power of the designed TX front-end. The saturated power P_sat_ of the TX at 24 GHz exceeds 11.7 dBm, as illustrated. The peak PAE is better than 47%. It is evident from this high-efficiency result that the presented TX architecture is feasible for automotive radars.

**Figure 13:**
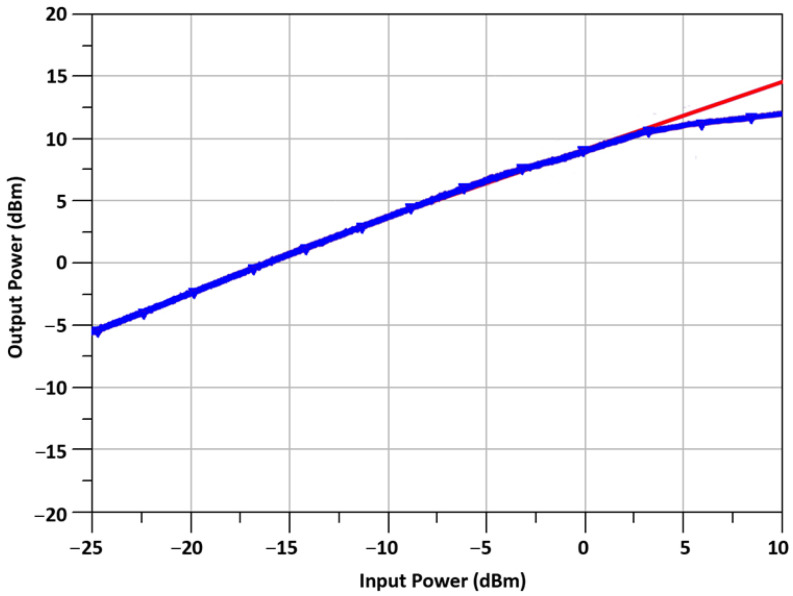
TX output power versus IF port input power.

In Figure 13, the measured output power of the TX is plotted against the input power at the mixer’s IF port. According to our results, the output-referred 1 dB compression points (OP_1_dB) for an IF of 2.4 GHz and an LO of 21.6 GHz were 10.5 dBm. Approximately 11.7 dBm is the saturated power of the TX.

Table 3 and Table 4 show the measured results for the TX front-end and performance comparison for the TX front-end, respectively.

## 4. Conclusions and Future Research

In conclusion, the systematic study of the 24 GHz TX front-end for radar applications represents a significant contribution to the field of radar technology. This study’s findings and insights offer an understanding of the challenges and opportunities associated with 24 GHz radar systems. This study addresses the growing demand for advanced radar technology operating in the 24 GHz frequency band. The TX front-end design incorporates an up-conversion mixer and a PA. The up-conversion mixer, based on a Gilbert cell, plays a crucial role in translating the 2.4 GHz intermediate frequency (IF) signal and the 21.6 GHz LO signal to the desired 24 GHz RF output signal. The integrated PA in the TX front-end is a class AB tunable two-stage PA. The use of varactors enables tuning in a synchronous mode to increase the PA gain. The use of 65 nm CMOS technology highlights the potential for achieving a high performance in radar applications. An evaluation of the TX front-end shows a conversion gain (CG) of 28.1 dB. Also, the input reflection coefficient of S_11_ is −13.2 dB, while the output reflection coefficient of S_22_ is −18.7 dB. It was demonstrated that the proposed TX front-end has a P_sat_of 11.7 dBm. In addition, there is a high PAE of 47%. A high-linearity OP_1_dB of 10.5 dBm at 24 GHz. The proposed TX front-end design showcases the potential for an enhanced performance, marked by improvements in power efficiency and linearity. The methodologies, insights, and innovative designs put forth in this research serve as a valuable resource for researchers to enhance the performance and capabilities of 24 GHz radar systems, furthering their application across a wide range of industries.

This research opens up several promising future research directions in the field of radar system design and RF front-end technology, such as investigating the design and analysis of RF front-ends in the context of multifunction radar systems. These systems perform various tasks, like target detection, tracking, and communication, simultaneously. Research may focus on how to design front-ends that are versatile and adaptable to these multifaceted demands. Also, how machine learning algorithms can be integrated with the RF front-end to optimize signal processing, target recognition, and the overall performance of radar systems could be investigated. This could lead to more intelligent and adaptive front-end systems. Front-end design strategies for miniaturization and integration in compact and portable radar systems, such as drone-based radar and IoT sensors, could be explored. These future research scope areas could help advance the field of radar technology, making radar systems more versatile, efficient, and capable of addressing the evolving needs of various applications in areas like defense, autonomous vehicles, environmental monitoring, and beyond.

## Figures and Tables

**Table 1:** Designed mixer circuit component values.

Parameter	Dimension
M1∼ M_2_	36 μm/65 nm
M_3_ ∼M_4_	64 μm/65 nm
M5∼ M_6_	36 μm/65 nm
M_7_ ∼ M_8_	216 μm/65 nm
M_9_ ∼ M_12_	40 μm/65 nm
M_Pb_ ∼ M_Nb_	90 μm/65 nm
L_1_ ∼ L_2_	180 pH
L_d1_ ∼ L_d2_	250 pH
L_s1_ ∼ L_s2_	150 pH
R_1_	2 KΩ
R_f_	4 KΩ
C_B_	80 pF
C_B1_ ∼ C_B2_	145 pF

**Table 2:** Designed PA circuit component values.

Parameter	Dimension
L_1_	0.65 nH
L_2_	0.78 nH
L_3_	1.7 nH
L_4_	0.91 nH
L_5_	1.1 nH
L_6_	1.8 nH
L_7_	1.5 nH
R1∼R_2_	73 Ω
R3∼R_4_	78 Ω
C1∼C3∼C_7_	0.5 pF
C_2_	0.87 pF
C_4_	0.51 pF
C_5_	0.37 pF
C_6_	0.41 pF
C__v1__	0.45 pF
C__v2__	0.2 pF

**Table 3:** Measured results for the TX front-end.

**Parameter**	Supply voltage Technology RF frequency IF frequency	1.2 V 65 nm CMOS 24 GHz 2.4 GHz
**Up-Conversion Mixer**	Conversion gain Noise figure OP_1_dB Power consumption Chip area	2.49 dB 3.9 dB 3.9 dBm 3.24 mW 0.42 mm^2^
**Power Amplifier**	Conversion gain IIP3 PAE P_sat_ Chip area	28.4 ± 0.5 dB 14.5 dBm 47.5% 14.21 dBm 0.406 mm^2^
**TX RF Front-End**	Conversion gain S_11_/S_22_ OP_1_dB PAE P_sat_ Chip area	28.1 dB −13.2/−18.7 dB 10.5 dBm 47% 11.7 3.2 mm^2^

**Table 4:** Performance comparison for the TX front-end.

Parameters	This Work	[26]	[27]	[28]	[29]
Technology	65	65	90	65	40
Freq. (GHz)	24	26	60	60	60
PAE (%)	47	N/A	N/A	N/A	N/A
P_DC_ (mW)	7.5	267	230	168	217
OP_1_dB (dBm)	10.5	10	3.7	N/A	N/A
P_sat_ (dBm)	11.7	14.1	8	10.3	15.6
Chip area (mm^2^)	3.2	2.31	37.4	1.03	0.33

## Data Availability

Data are contained within the article.
